# Evaluation of treatment outcomes of zygomaticomaxillary complex (ZMC) fractures using Foley catheter ballooning technique

**DOI:** 10.3389/fdmed.2026.1843337

**Published:** 2026-07-27

**Authors:** Rounak Mukherjee, Tripthi P. Shetty, Padmaraj J. Hegde

**Affiliations:** Department of Oral and Maxillofacial Surgery, AB Shetty Memorial Institute of Dental Sciences (ABSMIDS), Nitte (Deemed to be University), Mangalore, India

**Keywords:** anterior wall of maxillary sinus, Foley catheter, n-butyl cyanoacrylate glue, ORIF, ZMC fractures

## Abstract

**Introduction:**

Zygomaticomaxillary complex (ZMC) fractures are among the most common midfacial injuries. The conventional management involves open reduction and internal fixation (ORIF), which allows precise anatomical reduction but is associated with increased surgical morbidity. To overcome these limitations, minimally invasive techniques have been explored. The Foley catheter ballooning technique has recently been introduced as a novel method for fracture reduction. In addition, n-butyl cyanoacrylate glue has been used as an adjunct for fixation, offering advantages such as ease of application, reduced operative time, and elimination of hardware-related complications.

**Methodology:**

A prospective comparative study was conducted on 42 patients, allocated into two groups: Group A (Foley catheter ballooning technique, *n* = 21) and Group B (conventional ORIF, *n* = 21). Parameters evaluated included operative time, ease of procedure, postoperative stability, radiological outcomes (fracture reduction, ZMC and zygomatic arch symmetry, and orbital rim continuity), pain perception, and postoperative complications. Statistical analysis was performed using SPSS version 26. Variables were analyzed using the unpaired t-test and Chi-square test, with *p* < 0.05 considered statistically significant.

**Results:**

The mean operative time was significantly shorter in Group A compared with Group B (*p* = 0.001). Ease of procedure, postoperative stability, and radiological outcomes were significantly better in Group A. Pain perception and postoperative complications were also significantly lower in the Foley catheter group.

**Conclusion:**

The Foley catheter ballooning technique appears to be a reliable and minimally invasive alternative for selected ZMC fractures, providing reduced operative time, improved stability and better patient comfort compared with conventional ORIF.

## Introduction

Maxillofacial trauma represents a significant proportion of emergency surgical practice owing to the exposed anatomical position and complex functional role of the facial skeleton, and among midfacial injuries, fractures of the zygomaticomaxillary complex (ZMC) are particularly prevalent due to the prominence of the malar region and its vulnerability to traumatic forces. The zygomatic bone is essential for maintaining facial contour, protecting the globe, facilitating mastication, and contributing to midfacial stability; consequently, disruption of the ZMC frequently results in both functional impairment and aesthetic deformity, necessitating accurate diagnosis and appropriate management (1). Anatomically, the ZMC is a tetrapod structure articulating with the frontal, temporal, maxillary, and sphenoid bones, and fractures typically occur along these articulations, leading to displacement of the malar eminence, infraorbital rim, zygomatic arch, and lateral orbital wall depending on the magnitude and direction of the applied traumatic force (1). Clinically, ZMC fractures commonly present with facial asymmetry, malar flattening, periorbital edema or ecchymosis, infraorbital nerve paresthesia, diplopia, trismus, and restricted mandibular movements secondary to coronoid impingement (2). The etiology of ZMC fractures varies geographically, with road traffic accidents predominating in developing countries, whereas interpersonal violence and sports-related injuries are more common in developed nations, and accurate diagnosis relies on careful clinical examination supported by appropriate imaging (2). The primary objectives in managing ZMC fractures are precise anatomical reduction, stable fixation, restoration of facial symmetry, and prevention of functional deficits, and although open reduction and internal fixation (ORIF) remains the standard approach for displaced fractures, it may be associated with increased surgical morbidity (1). Consequently, minimally invasive adjunctive techniques have gained interest, and the Foley catheter ballooning technique provides controlled internal support through the maxillary sinus, facilitating elevation and stabilization of displaced fracture segments, particularly in fractures involving the zygomatic arch and the anterior wall of the maxillary sinus (3). Recent clinical evidence also suggests that N-butyl-2-cyanoacrylate glue can be effectively utilized for stabilization of comminuted fractures of the anterior wall of the maxillary sinus, offering advantages such as ease of application, reduced operative time, and avoidance of hardware-related complications while achieving satisfactory clinical outcomes (4). Therefore, the present study aims to evaluate the clinical and radiological outcomes of zygomaticomaxillary complex fractures managed using the Foley catheter ballooning technique as a minimally invasive adjunct for fracture stabilization (1–4).

## Materials and methods

### Study design and ethical approval

This prospective, comparative clinical study included 42 patients diagnosed with zygomaticomaxillary complex (ZMC) fractures requiring open reduction and internal fixation (ORIF). Patients were recruited from Justice K.S. Hegde Charitable Hospital and A.B. Shetty Memorial Institute of Dental Sciences between December 2023 and December 2025. This study was conducted in accordance with the ethical principles outlined in the Declaration of Helsinki for research involving human participants. Ethical approval was obtained from the Institutional Ethics Committee of A.B. Shetty Memorial Institute of Dental Sciences, Nitte (Deemed to be University), Mangalore, India (Approval No.: ETHICS/AB SHETTY MEMORIAL INSTITUTE OF DENTAL SCIENCE/431/2024). Written informed consent was obtained from all participants prior to enrolment in the study. Participation was voluntary, and all patient information was kept confidential and used solely for research purposes.

### Patient selection

Eligible participants were adults between age 18−65years of either sex presenting with unilateral ZMC fractures, minimally displaced fracture of anterior wall of maxillary sinus and comminuted fracture of ZMC involving floor of the orbit indicated for ORIF. All fractures were classified preoperatively according to the Zingg classification system based on CT/CBCT findings. Only patients presenting with Type B (complete ZMC fractures) and selected Type C (comminuted ZMC fractures involving the orbital floor without orbital fat herniation) were included in the study. Patients were randomly allocated into two equal groups using a lottery method: Experimental Group A (*n* = 21): ZMC fracture reduction using the Foley catheter ballooning technique and Control Group B (*n* = 21): ZMC fracture reduction using the conventional surgical technique. This randomisation procedure was employed to minimise selection bias and ensure comparable allocation between the study groups Exclusion criteria included previous treatment for facial fractures, cases with fat herniation after traumatic injury to orbit, syndromic conditions, debilitating systemic disease, psychological disorders, refusal to provide informed consent, and inability or unwillingness to comply with postoperative follow-up.

### Surgical protocol

**Figure 1:**
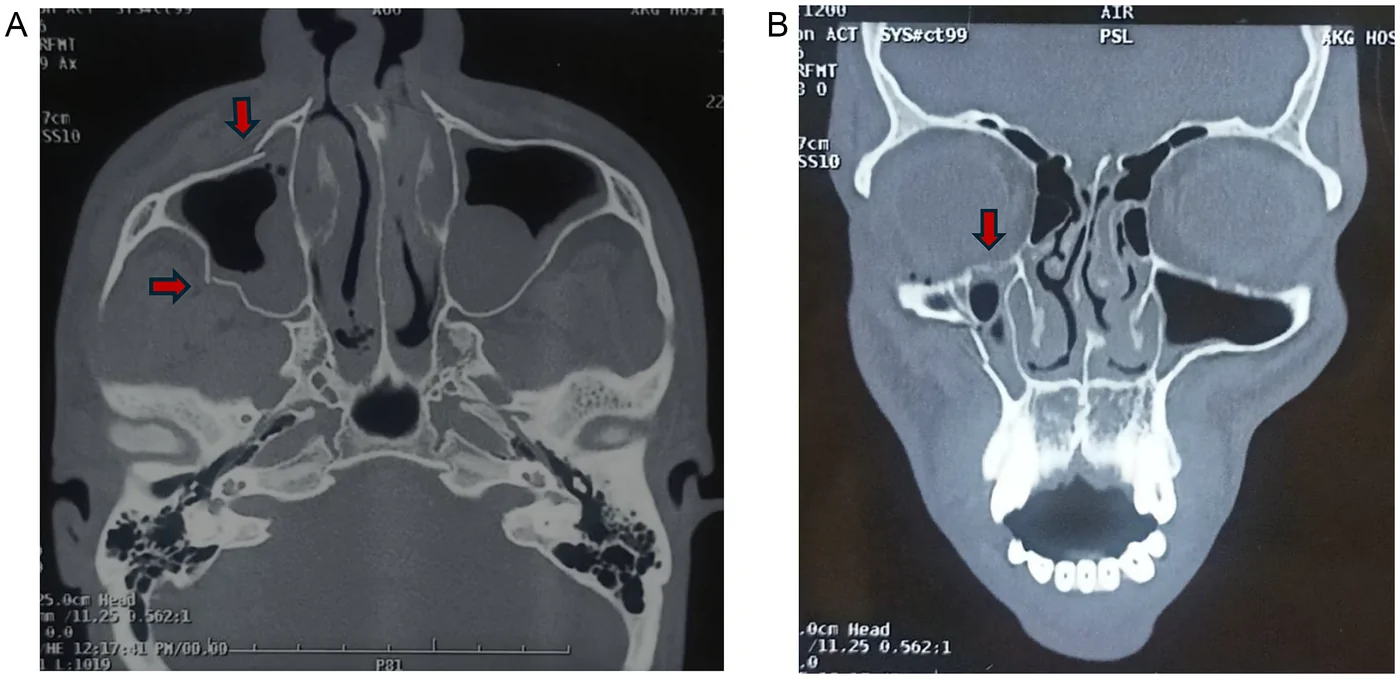
**(A)** Preoperative CT scan (axial view). **(B)** Preoperative CT scan (coronal view).

All procedures were performed under general anaesthesia with nasotracheal intubation, following strict aseptic protocols, by two qualified oral and maxillofacial surgeons with appropriate postgraduate training and ten years clinical experience, ensuring consistency in surgical technique and optimal patient outcomes. Patients who were included in the study had undergone preoperative CT/CBCT scan (Figures 1A,B).

### Fracture reduction using Foley catheter ballooning technique (experimental group A)

**Figure 2:**
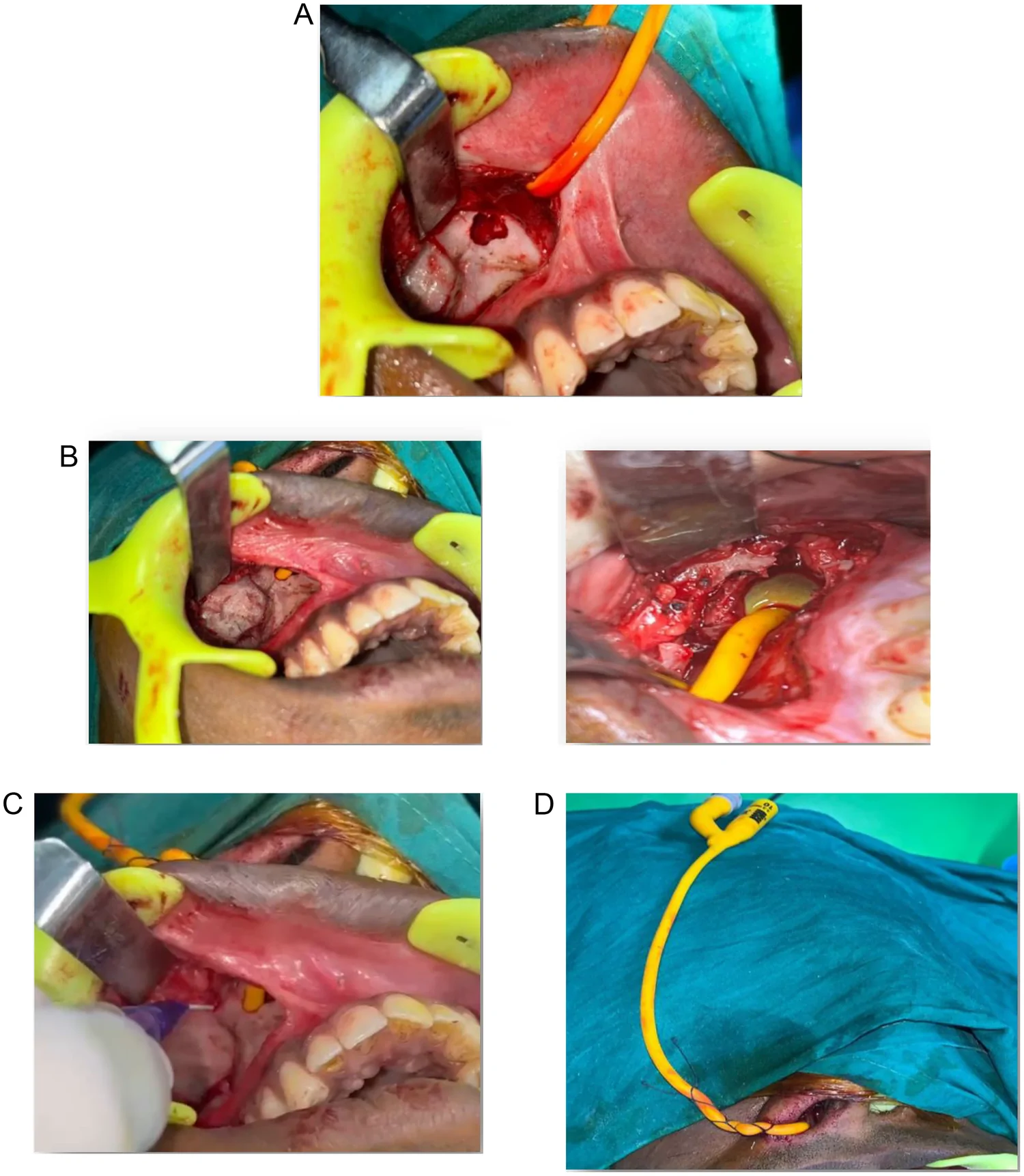
**(A)** The Foley catheter was introduced to the ZMC fracture site through fractured segments. **(B)** The balloon end of the catheter was introduced into the site of fracture and inflated by using sterile saline. **(C)** The fractured segments were stabilized using n-butyl cyanoacrylate glue. **(D)** The free end of the catheter was secured extraorally through nasal opening.

Following standard aseptic preparation and draping, a maxillary buccal vestibular incision extending from the canine to the first molar region was placed. A full-thickness mucoperiosteal flap was elevated to expose the zygomaticomaxillary buttress and anterior wall of the maxillary sinus. In cases where the anterior maxillary wall fracture provided adequate access, a 14–16 Fr Foley catheter was introduced through the existing fracture defect into the maxillary sinus. When adequate access was not available, a small bony window (approximately 1–1.5 cm) was created in the anterior wall of the maxillary sinus (Figure 2A). The purpose of this window was to facilitate controlled insertion of the catheter and accurate positioning of the balloon beneath the displaced ZMC segment, thereby enabling effective fracture elevation and stabilization. The window was kept as small as possible to minimize additional surgical trauma and was created within the already exposed operative field without requiring an additional external incision. As the anterior maxillary wall was frequently involved in the fracture pattern and subsequently stabilized during the procedure, creation of this limited access window was not considered to significantly increase operative morbidity or adversely affect postoperative healing. The balloon portion of the catheter was positioned beneath the displaced ZMC segment under direct visualization. The balloon was gradually inflated with 10–20 mL sterile normal saline until satisfactory elevation and anatomical reduction of the zygomatic complex was achieved (Figure 2B). Reduction was confirmed clinically by restoration of malar projection and alignment of the zygomaticomaxillary buttress and, where indicated, by intraoperative imaging. Following reduction, comminuted anterior maxillary wall fragments were stabilized using N-butyl-2-cyanoacrylate adhesive applied directly over the reduced bony segments (Figure 2C). The balloon remained inflated to provide internal support during polymerization of the adhesive. The surgical site was irrigated with sterile saline and closed using 4-0 polyglactin (Vicryl) sutures. The catheter was secured and maintained *in situ* for two weeks, with the distal end exteriorized through a small nasal opening. The catheter balloon was gradually deflated and removed after two weeks (Figure 2D).

### Fracture reduction using conventional technique (control group B)

**Figure 3:**
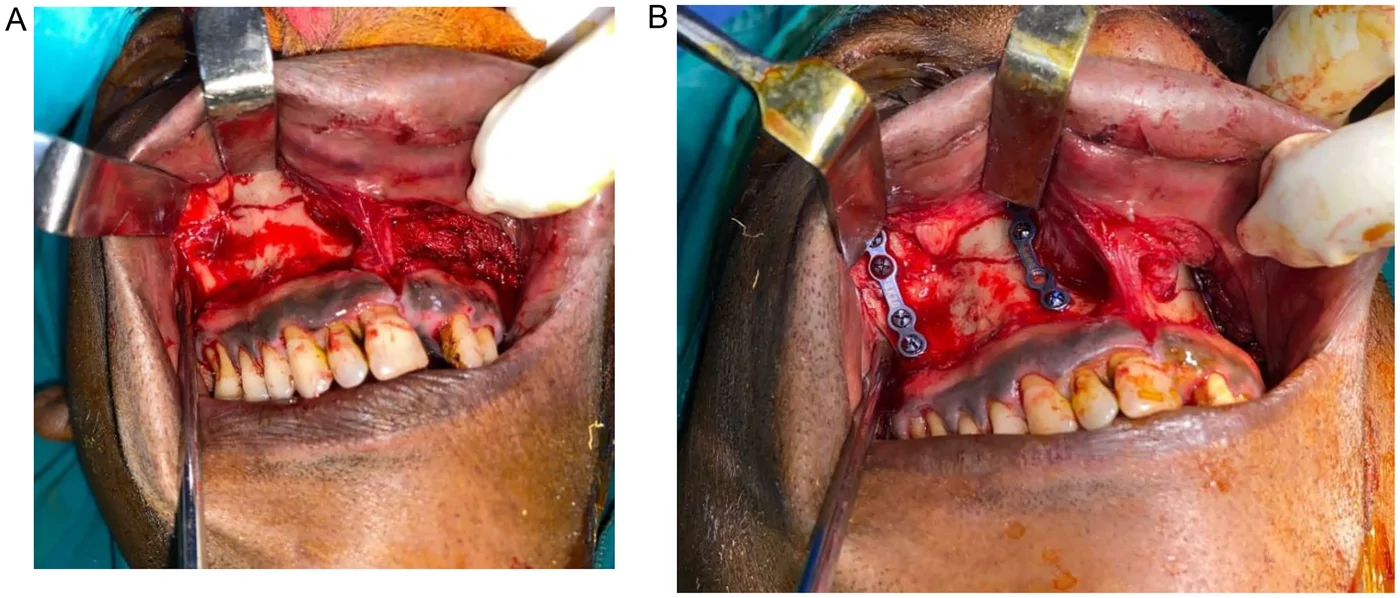
**(A)** Exposed fracture fragment site after reduction. **(B)** Fixation of fracture segments with miniplates.

An intraoral buccal vestibular incision was placed to expose the fracture site, followed by elevation of a full-thickness mucoperiosteal flap. Reduction was achieved by manual manipulation using appropriate elevators. A zygomatic elevator was positioned beneath the displaced zygomatic body, and controlled upward, forward, and outward forces were applied to restore anatomical alignment. Stable fixation was achieved using titanium miniplates (1.5-mm or 2.0-mm) and screws. Associated facial fractures were managed simultaneously according to standard treatment protocols. (Figures 3A,B).

**Figure 4:**
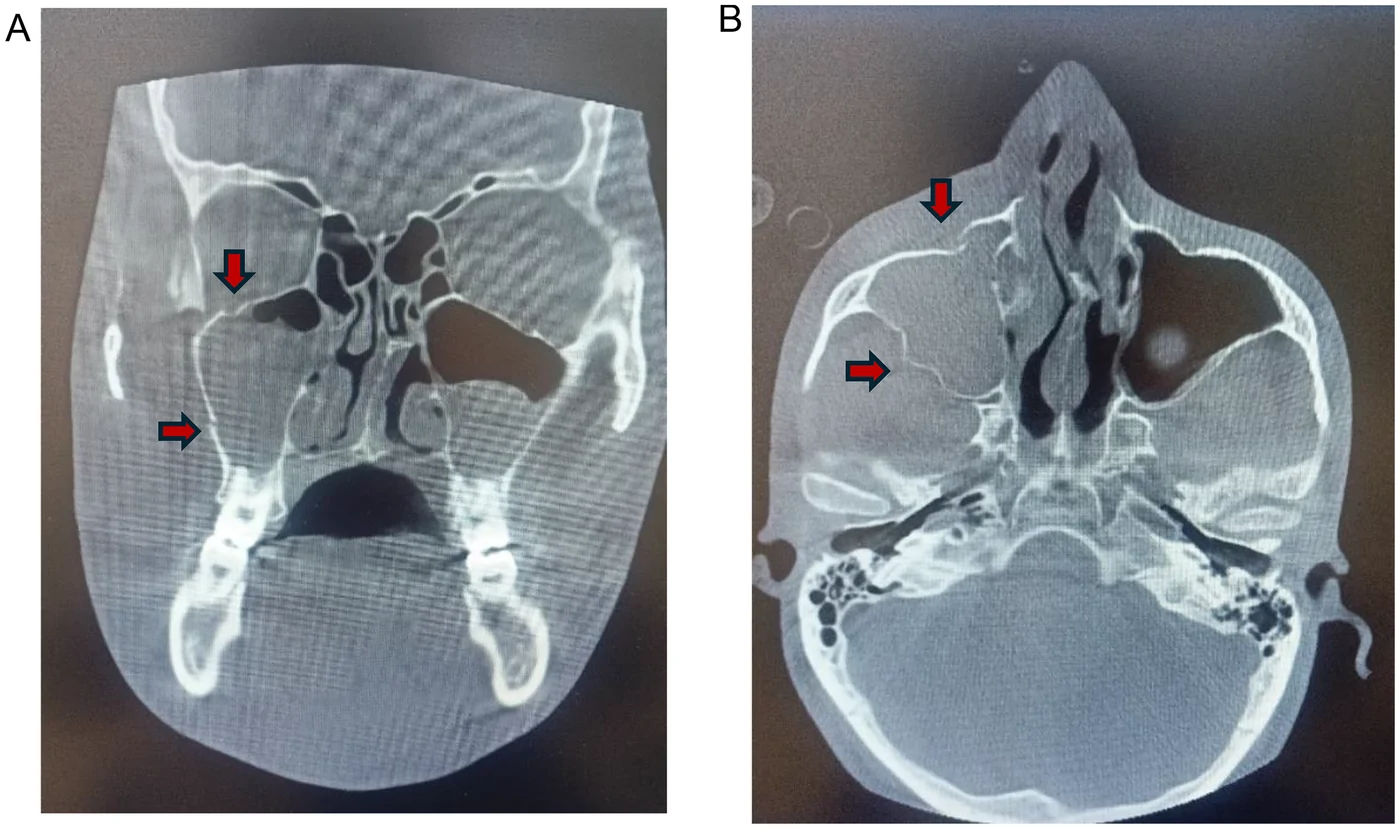
**(A)** immediate postoperative CT scan (coronal view). **(B)** Immediate postoperative CT scan (axial view).

**Figure 5:**
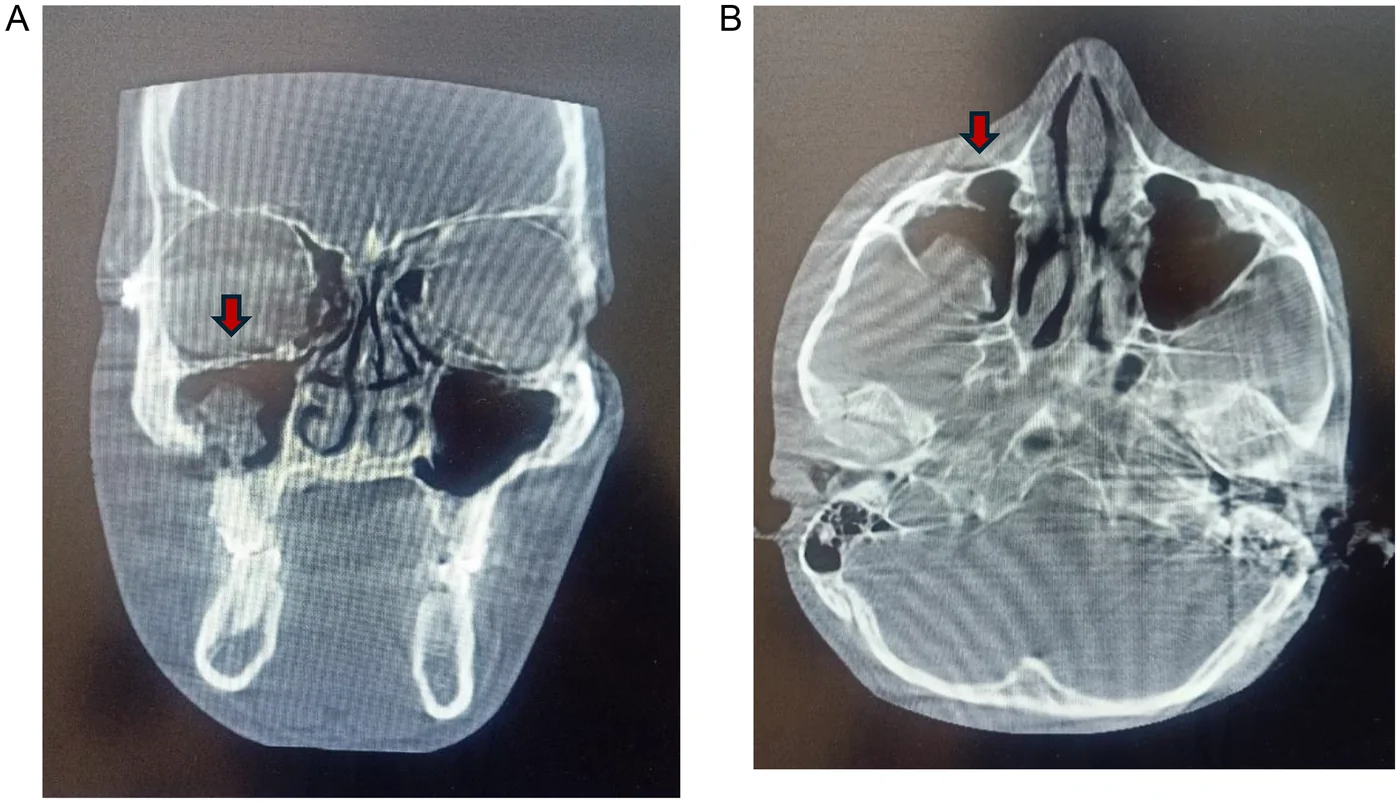
**(A)** 2 months postoperative CT scan (coronal view). **(B)** 2 months postoperative CT scan (axial view).

Postoperative CBCT or CT imaging was obtained to assess postoperative reduction accuracy in both groups (Figures 4A,B). Follow-up CBCT/CT imaging was performed at two months postoperatively to evaluate fracture healing and stability (Figures 5A,B). Both of the groups were reviewed clinically during follow-up visits of 6 months for assessment of stability, symmetry of zygomatic arch, continuity of orbital rim, pain and any other postoperative complications.

### Assessments of parameters and treatment outcomes

The time required for the procedure was assessed in seconds using a standardized stopwatch and was calculated from the initiation of fracture reduction until fixation of the fracture segments using the Foley catheter ballooning technique followed by fixation with N-butyl-2-cyanoacrylate glue for Group A and fixation of miniplates for Group B. The ease of the procedure was evaluated immediately after reduction of the zygomaticomaxillary complex (ZMC) fracture in both the Groups from the surgeon's perspective using a Quality Likert Scale ranging from 1 to 5, where 1 indicated extremely difficult and 5 indicated extremely easy. The adequacy of intraoperative reduction of the ZMC fracture was assessed after reduction of the fractured fragments from the surgeon's perspective using a nominal scale and recorded as satisfactory (1) or unsatisfactory (0). Postoperative stability of fracture fixation was assessed at 2 weeks and 2 months following surgery by clinical digital palpation, which involved applying alternating manual pressure across the fractured segments, and the findings were recorded using a nominal scale as stable (1) or unstable (0). Fracture reduction and postoperative stability were further evaluated using CBCT or CT imaging. Postoperative imaging was obtained 2 weeks following surgery to assess the adequacy of fracture reduction, while follow-up CBCT/CT imaging was performed at 2 months postoperatively to evaluate maintenance of reduction, fracture healing, and postoperative stability. The radiological assessment included evaluation of fracture segment alignment, restoration of ZMC and ZA symmetry relative to the contralateral unaffected side, continuity of the infraorbital/orbital rim, and the presence or absence of secondary displacement during follow-up. Fracture reduction was categorized as satisfactory when anatomical alignment, facial symmetry, and orbital rim continuity were restored without clinically significant displacement. Cases demonstrating residual displacement, asymmetry, or disruption of orbital rim continuity were categorized as unsatisfactory. Postoperative complications were also assessed, and pain was evaluated using the Visual Analog Scale (VAS, a validated pain assessment tool consisting of a 10-cm horizontal line, where 0 represented no pain and 10 represented the worst pain imaginable) on postoperative days 1, 7, and 14 following completions of ZMC fracture treatment, while any additional postoperative complications, if present, were documented and analysed.

## Statistical analysis

The sample size was calculated based on a comparison of proportions between two groups. The proportion in Group A was assumed to be 0.07 and in Group B as 0.10, with an estimated risk difference of 0.03 and a population risk difference of 0.10. Based on these assumptions, the required sample size was calculated to be 42 participants. With a 90% confidence level, the sample size was estimated to be 21 participants in each group.

The data was analysed using SPSS for Windows [Ver 26.0, IBM Corp., Armonk, NY]. Continuous data was compared between the group using the unpaired t-test and categorical data was compared between the groups using the Chi-square test. Results were presented using tables and graphs. The level of significance was set at *p* < 0.05.

## Results

**Figure 6:**
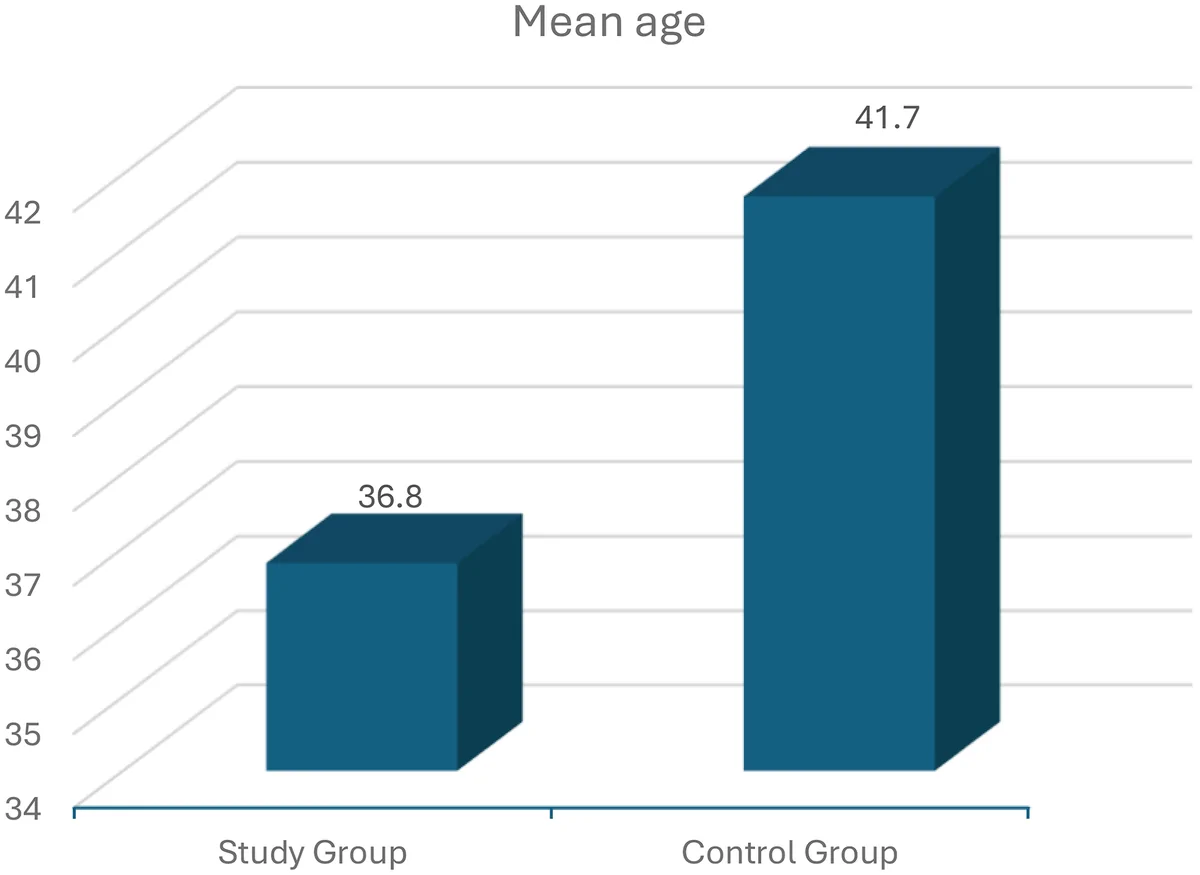
Mean age of participants in both groups.

**Figure 7:**
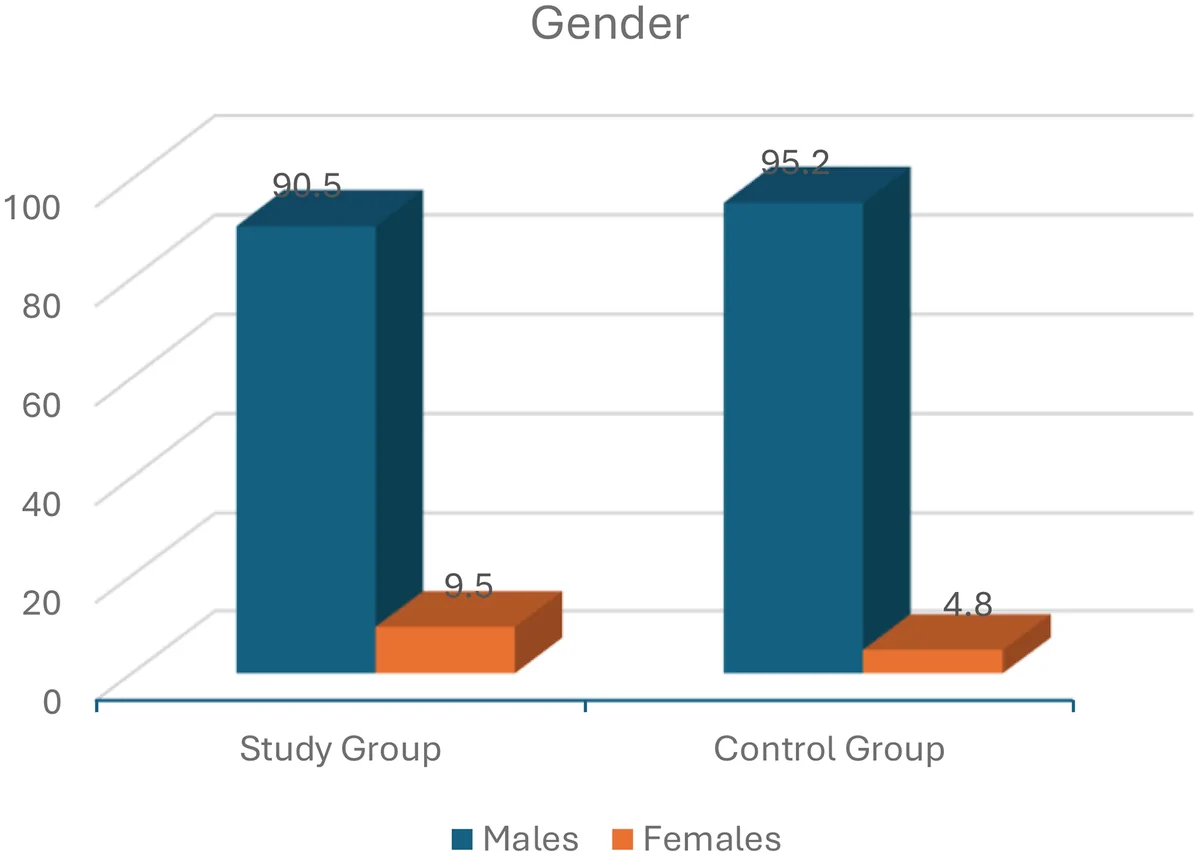
Distribution of participants according to gender.

A total of 42 patients were included in the study and equally allocated into Experimental Group A (Foley catheter ballooning technique; *n* = 21) and Group B (conventional surgical technique; *n* = 21). The mean age of the patients was 36.8 ± 12.6 years in Group A and 41.7 ± 16.9 years in Group B, with no statistically significant difference between the groups (*p* = 0.29, CI = 95%) (Table 1, Figure 6). A clear male predominance was observed in both groups, with Group A consisting of 2 female (9.5%) patients and 19 male (90.5%) patients, while Group B included 1 female (4.8%) and 20 male (95.2%) patients (*p* = 0.54) (Table 2, Figure 7).

**Table 1:** Comparison of mean age of participants between the groups.

Group	Number	Mean	SD	t	*p* value
Study group	21	36.8	12.6	−1.06	*p* = 0.29
Control group	21	41.7	16.9		NS

SD, standard deviation; NS, not significant using the unpaired t-test; CI- 95%.

**Table 2:** Distribution of participants according to gender.

	Study Group		Control Group		Chi value	*p* value
Gender	Number	Percentage	Number	Percentage		
Males	19	90.5	20	95.2	0.36	*p* = 0.54
Females	2	9.5	1	4.8		NS
Total	21	100	21	100		

NS, not significant using the Chi-square test.

**Figure 8:**
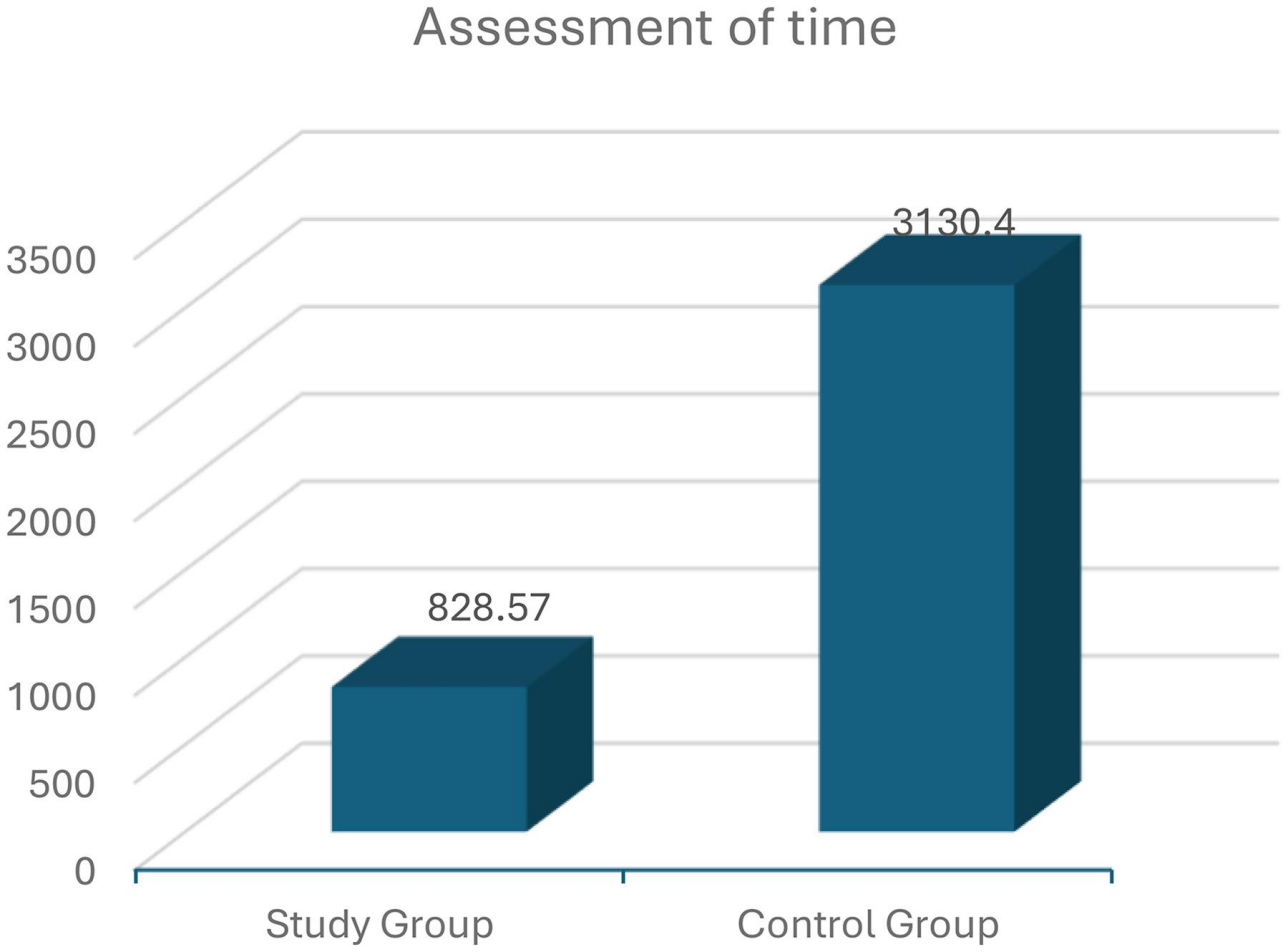
Mean time taken to complete the procedure (in seconds) in both groups.

**Figure 9:**
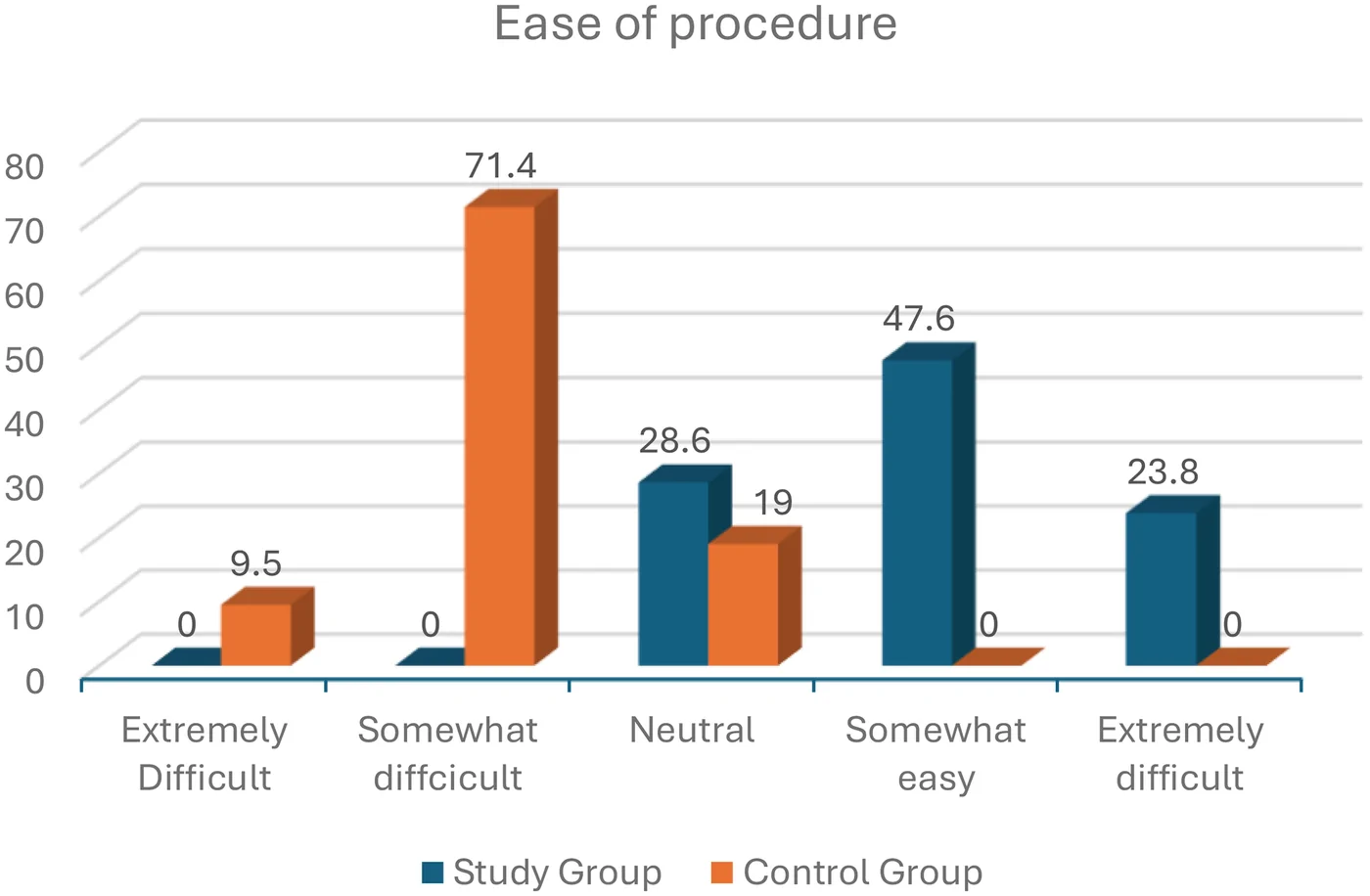
Distribution according to ease of procedure between the groups.

The mean operative time was significantly shorter in Group A (828.57 ± 145.2 s) compared with Group B (3,130.4 ± 718.01 s), and unpaired t-test analysis demonstrated a statistically significant difference between the groups (t = −14.4, *p* = 0.001) with a mean difference of 2,301.83 s (95% CI) (Table 3, Figure 8). Evaluation of procedural ease also revealed a significant intergroup difference (*χ*^2^ = 32.4, *p* = 0.001). Most procedures in Group B were rated as somewhat difficult (71.4%) or extremely difficult (9.5%), whereas no procedures in Group A were reported as difficult. In contrast, 47.6% and 23.8% of procedures in Group A were rated as somewhat easy and extremely easy respectively, while 28.6% of Group A and 19.0% of Group B cases were rated as neutral (Table 4, Figure 9).

**Table 3:** Comparison of mean time taken to complete the procedure (in seconds) between the groups.

Group	Number	Mean	SD	t	*p* value
Study group	21	828.57	145.2	−14.4	*p* = 0.001*
Control group	21	3,130.4	718.01		

SD, standard deviation;

* statistically significant using the unpaired t-test; CI-95%.

**Table 4:** Distribution according to ease of procedure between the groups.

	Study group		Control group		Chi value	*p* value
Scale	Number	Percentage	Number	Percentage		
Extremely difficult	0	0	2	9.5	32.4	*p* = 0.001*
Somewhat difficult	0	0	15	71.4		
Neutral	6	28.6	4	19		
Somewhat easy	10	47.6	0	0		
Extremely easy	5	23.8	0	0		
Total	21	100	21	100		

* Statistically significant using the Chi-square test.

**Figure 10:**
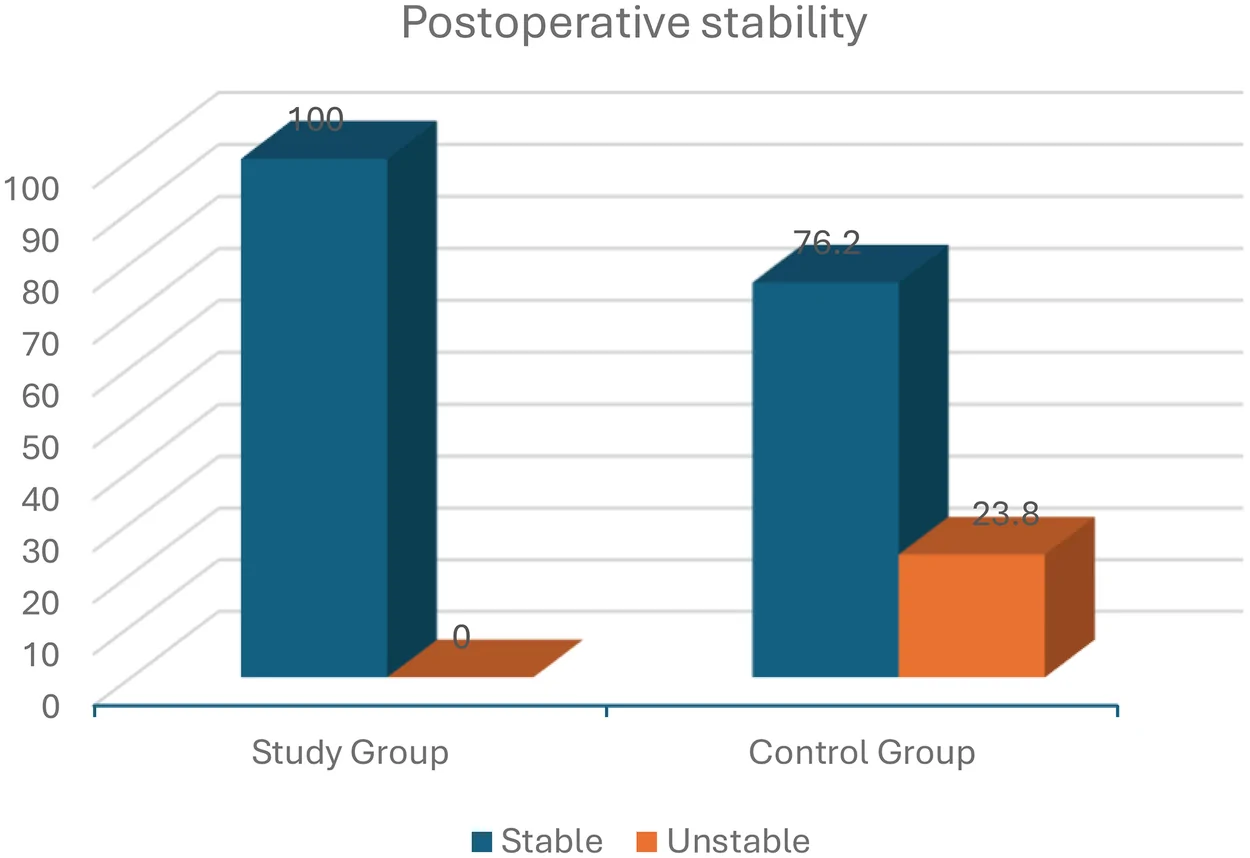
Postoperative stability of procedures between the groups.

**Figure 11:**
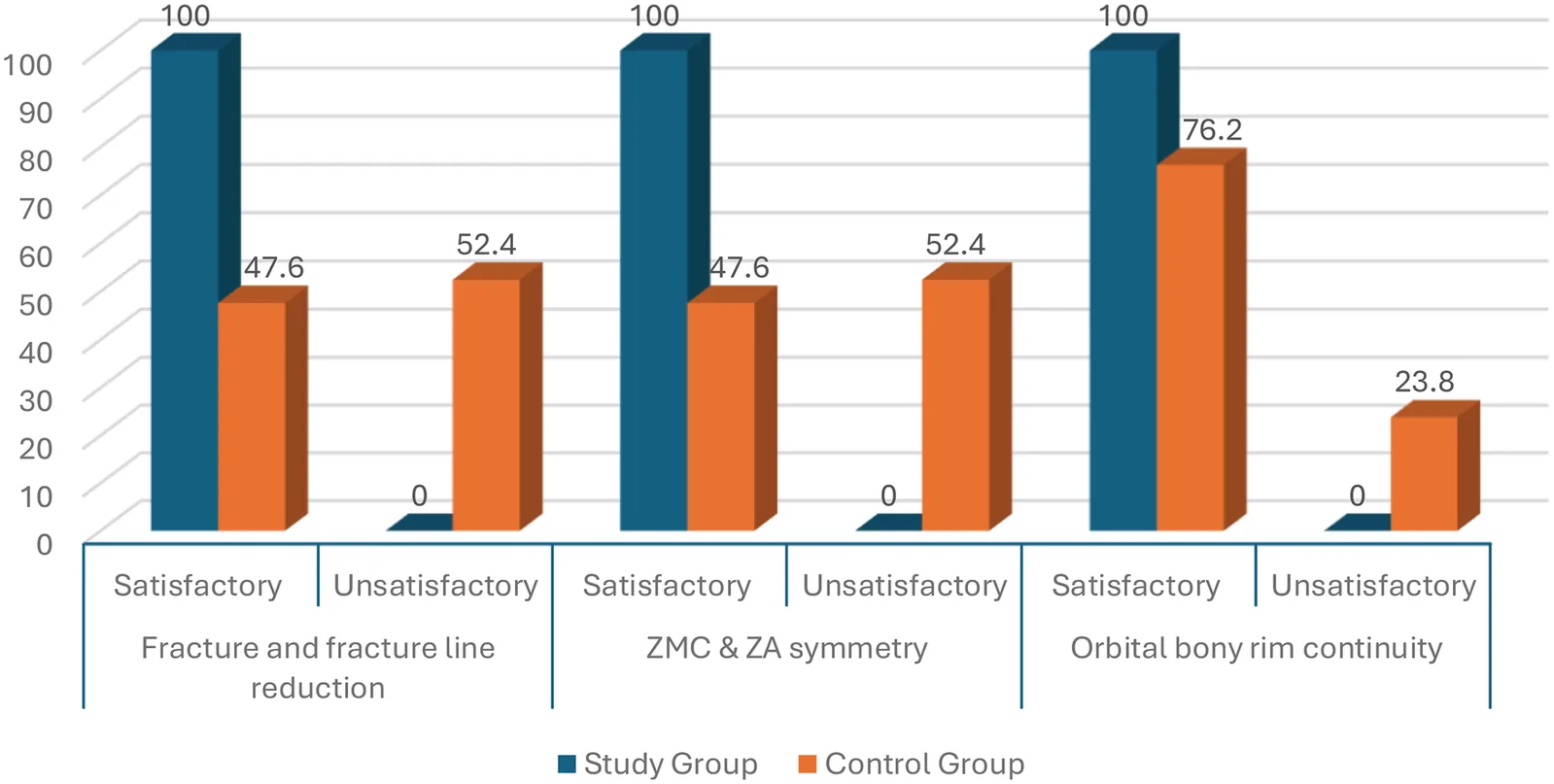
Distribution of participants according to reduction of fracture & fracture lines, symmetry of ZMC and ZA, and continuity of orbital bony rim between the groups.

**Figure 12:**
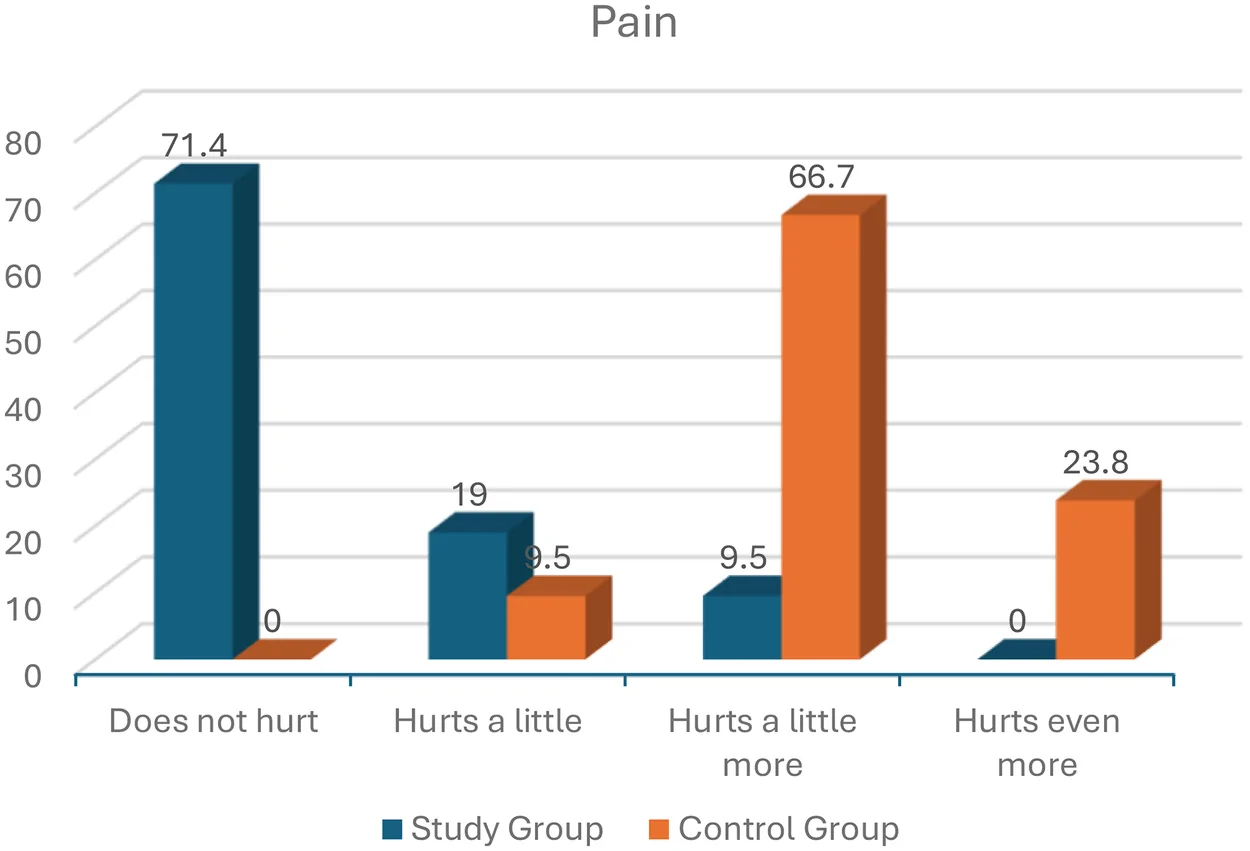
Distribution of participants according to pain between the groups.

**Figure 13:**
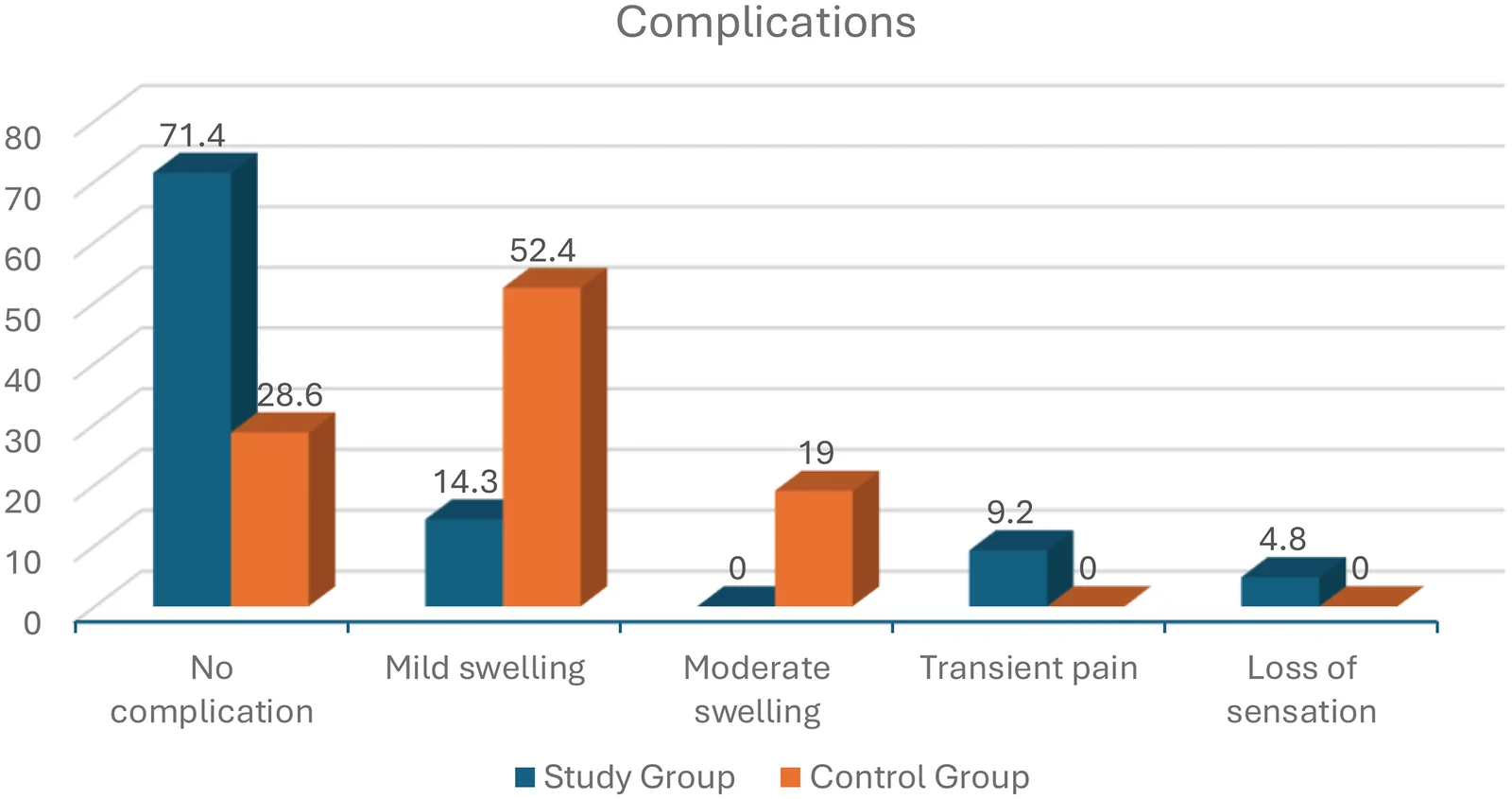
Distribution of participants according to complications between the groups.

Postoperative stability was achieved in all patients in Group A (100%), whereas stability was observed in 76.2% of patients in Group B, with instability noted in 23.8% of cases. This difference was statistically significant (*χ*^2^ = 5.67, *p* = 0.048) (Table 5, Figure 10). Radiological assessment demonstrated satisfactory fracture reduction and restoration of ZMC and ZA symmetry in all patients in Group A (100%) compared with 47.6% in Group B (*χ*^2^ = 14.9, *p* = 0.001). Similarly, orbital rim continuity was satisfactory in all patients in Group A and in 76.2% of patients in Group B (*χ*^2^ = 5.67, *p* = 0.048) (Table 6, Figure 11). Patient-reported pain scores also showed a significant difference between groups (*χ*^2^ = 29.6, *p* = 0.001). In Group A, 71.4% of patients reported no pain and 19.0% reported mild pain, whereas most patients in Group B experienced higher pain levels, with 66.7% reporting moderate pain and 23.8% reporting severe pain. (Table 7, Figure 12). Postoperative complications were significantly fewer in Group A (*χ*^2^ = 15.4, *p* = 0.004). No complications were observed in 71.4% of patients in Group A compared with 28.6% in Group B, while mild and moderate postoperative swelling were more common in Group B (52.4% and 19.0%, respectively). Transient pain (9.2%) and temporary sensory disturbance (4.8%) were observed only in Group A (Table 8, Figure 13). Table 9 shows summary of comparison of both the groups based on clinical and radiological outcomes.

**Table 5:** Distribution of participants according to postoperative stability between the groups.

	Study group		Control group		Chi value	*p* value
Stability	Number	Percentage	Number	Percentage		
Stable	21	100	16	76.2	5.67	*p* = 0.048*
Unstable	0	0	5	23.8		
Total	21	100	21	100		

* Statistically significant using the Chi-square test.

**Table 6:** Distribution of participants according to reduction of fracture & fracture lines, symmetry of ZMC and ZA, and continuity of orbital bony rim between the groups.

		Study group		Control group		Chi value	*p* value
Reduction	Scale	Number	Percentage	Number	Percentage		
Fracture and fracture line reduction	Satisfactory	21	100	10	47.6	14.9	*p* = 0.001*
	Unsatisfactory	0	0	11	52.4		
	Total	21	100	21	100		
ZMC & ZA symmetry	Satisfactory	21	100	10	47.6	14.9	*p* = 0.001*
	Unsatisfactory	0	0	11	52.4		
	Total	21	100	21	100		
Orbital bony rim continuity	Satisfactory	21	100	16	76.2	5.67	*p* = 0.048*
	Unsatisfactory	0	0	5	23.8		
	Total	21	100	21	100		

ZMC, zygomatico-maxillary complex; ZA, zygomatic arch.

* Statistically significant using the Chi-square test.

**Table 7:** Distribution of participants according to pain between the groups.

	Study group		Control group		Chi value	*p* value
Pain score	Number	Percentage	Number	Percentage		
Does not hurt	15	71.4	0	0	29.6	*p* = 0.001*
Hurts a little	4	19	2	9.5		
Hurts a little more	2	9.5	14	66.7		
Hurts even more	0	0	5	23.8		
Total	21	100	21	100		

* Statistically significant using the Chi-square test.

**Table 8:** Distribution of participants according to complications between the groups.

	Study group		Control group		Chi value	*p* value
Complication	Number	Percentage	Number	Percentage		
No complication	15	71.4	6	28.6	15.4	*p* = 0.004*
Mild swelling	3	14.3	11	52.4		
Moderate swelling	0	0	4	19		
Transient pain	2	9.2	0	0		
Loss of sensation	1	4.8	0	0		
Total	20	100	21	100		

* Statistically significant using the Chi-square test.

**Table 9:** Comparison of all the variables between the two groups.

Parameter	Group A (Foley catheter) *n* = 21	Group B (ORIF) *n* = 21	Test statistic	*p*-value
Age	36.8 ± 12.6	41.7 ± 16.9	t = −1.06	0.29
Gender *n* (%)	19 (90.5%)	20 (95.2%)	*χ*^2^ = 0.36	0.54
Operative time (seconds)	828.57 ± 145.2	3,130.4 ± 718.01	t = −14.4	0.001*
Postoperative stability *n* (%)	21 (100%)	16 (76.2%)	χ^2^ = 5.67	0.048*
Satisfactory fracture reduction *n* (%)	21 (100%)	10 (47.6%)	χ^2^ = 14.9	0.001*
Satisfactory ZMC & ZA symmetry *n* (%)	21 (100%)	10 (47.6%)	χ^2^ = 14.9	0.001*
Satisfactory orbital rim continuity *n* (%)	21 (100%)	16 (76.2%)	χ^2^ = 5.67	0.048*
No pain *n* (%)	15 (71.4%)	0 (0%)	χ^2^ = 29.6	0.001*
No postoperative complications, *n* (%)	15 (71.4%)	6 (28.6%)	χ^2^ = 15.4	0.004*

* Statistically significant using the Chi-square test.

## Discussion

The management of zygomaticomaxillary complex (ZMC) fractures aims to restore facial contour, achieve precise anatomical reduction, preserve ocular function, and minimize surgical morbidity. Conventional open reduction and internal fixation (ORIF) remains the standard treatment for displaced fractures; however, it often requires extensive surgical exposure and soft tissue dissection, which may increase operative time and postoperative morbidity, as described by Strong and Gary et al. (5) and Fonseca et al. (6) A detailed understanding of the surgical anatomy of the ZMC is essential for accurate fracture reduction and prevention of complications, as emphasized by Hwang and Kim et al. (7).

In the present study, inclusion criteria were limited to minimally displaced fractures of the anterior wall of the maxillary sinus associated with ZMC disruption and comminuted ZMC fractures involving the orbital floor requiring ORIF. Although isolated anterior maxillary wall fractures may sometimes be treated conservatively, their inclusion was justified when associated with ZMC fractures because even minor displacement can affect malar projection and midfacial symmetry. Additionally, fractures involving the orbital floor were included because disruption of orbital support may predispose to complications such as enophthalmos, diplopia, and infraorbital nerve dysfunction (8). Cases with orbital fat herniation were excluded because they require more complex orbital reconstruction procedures, which could introduce variability in surgical technique and outcomes (9). Although creation of a small anterior maxillary wall window was occasionally required to facilitate Foley catheter placement, this modification did not appear to increase postoperative morbidity in the present study. The window was created through the existing surgical exposure and was limited in size to minimize additional bone removal. Nevertheless, the theoretical risk of increased surgical trauma, postoperative discomfort, or delayed healing should be considered, particularly in patients with extensive comminution or compromised bone quality. Demographic characteristics in the present study were comparable between groups, which is consistent with epidemiological studies reporting male predominance and a wide age distribution in ZMC fractures Zingg et al. (10); Adeyemo et al. (11). A significant reduction in operative time was observed in the Foley catheter ballooning group. Previous studies have demonstrated that prolonged operative duration may increase perioperative complications such as infection, edema, and delayed recovery Cheng et al. (12); Kloss et al. (13). The reduced operative time observed in this study may be attributed to simplified fracture elevation and stabilization achieved using the Foley catheter technique, similar to findings reported by Bansal et al. (14) Greater intraoperative ease was also observed in the Foley catheter group, which supports the principle that surgical techniques minimizing tissue manipulation improve procedural efficiency, as described by Champy et al. (15) Postoperative stability was superior in the Foley catheter group, particularly in fractures involving non-weight-bearing regions of the midface. Ellis et al. (16) emphasized that adequate mechanical stability is essential to prevent secondary displacement and facial asymmetry. Internal support techniques may provide sufficient stabilization in fractures involving areas such as the anterior wall of the maxillary sinus or isolated zygomatic arch fractures. Similar stability with alternative fixation strategies has been reported by Kim et al. (17), while Manson et al. (18) highlighted that accurate anatomical reduction remains a key determinant of long-term functional and aesthetic outcomes. Radiological evaluation in the present study demonstrated improved fracture line reduction, restoration of ZMC and zygomatic arch symmetry, and maintenance of orbital rim continuity in the study group. Hammer et al. (19) reported that inadequate orbital reconstruction following ZMC fractures may lead to complications such as diplopia and enophthalmos. Minimally invasive approaches have also demonstrated improved anatomical outcomes with reduced morbidity, as described by Strong et al. (20) The present findings further support the effectiveness of Foley catheter ballooning in achieving satisfactory anatomical alignment with minimal surgical trauma. Postoperative pain was significantly lower in the study group. Tiwana (21) reported that postoperative discomfort following facial fracture surgery is closely related to operative duration and the extent of soft tissue dissection, while Pogrel (22) emphasized that reduced surgical trauma contributes to improved postoperative recovery. In addition, postoperative complications were fewer in the Foley catheter group. Plate and screw fixation may be associated with complications such as plate exposure, infection, hardware palpability, tactile discomfort, and soft tissue irritation. Modabber et al. (23) identified prolonged operative exposure and extensive tissue manipulation as important factors contributing to postoperative swelling, while Curtis et al. (24) reported that the degree of soft tissue response following ZMC repair is related to surgical exposure. Minor transient sensory disturbances observed in a few cases were self-limiting and did not affect overall outcomes. Long-term observations by Ellis et al. (25) suggest that minimizing early postoperative morbidity contributes to improved recovery and patient satisfaction. Indications include minimally displaced or moderately displaced ZMC fractures involving the anterior wall of the maxillary sinus, selected zygomatic arch fractures, and comminuted fractures of the anterior maxillary wall where conventional rigid fixation may be difficult or unnecessary. The technique may also serve as a minimally invasive adjunct to reduce operative time and soft tissue dissection.

However, this approach is not appropriate for all ZMC fractures. Contraindications include severely displaced fractures requiring rigid fixation, fractures with significant orbital disruption or orbital fat herniation, gross comminution resulting in loss of bony support, active maxillary sinus infection, and cases requiring extensive orbital reconstruction. In such situations, conventional open reduction and internal fixation remains the preferred treatment modality. Potential complications include balloon rupture or leakage, inadequate reduction, secondary displacement after catheter removal, transient infraorbital nerve paresthesia, postoperative pain, maxillary sinus irritation, sinusitis, infection, nasal discomfort related to the externalized catheter, and patient intolerance to prolonged catheter retention. Although no major complications were encountered in the present study, these risks should be considered during treatment planning. In rare situations, persistent malar asymmetry, residual displacement, or functional impairment may necessitate revision surgery and conversion to conventional open reduction and internal fixation.

Within the limitations of this study, the Foley catheter ballooning technique demonstrated favorable clinical and radiological outcomes in selected ZMC fractures, particularly those involving minimally displaced anterior maxillary wall fractures and comminuted fractures associated with the orbital floor. Compared with conventional ORIF, the technique was associated with reduced operative time, satisfactory fracture stability, improved radiological outcomes, lower postoperative pain, and fewer complications. Therefore, the Foley catheter ballooning technique may serve as a safe and minimally invasive adjunct in carefully selected cases of ZMC fractures. However, larger multicentric studies with longer follow-up periods are required to confirm its long-term efficacy and broader clinical applicability.

### Limitations

The present study had certain limitations. The sample size was relatively small and the follow-up period was limited to the early postoperative phase, restricting evaluation of long-term fracture stability, maintenance of midfacial symmetry, and delayed complications. Long-term infraorbital nerve recovery and radiological maintenance of fracture reduction were also not assessed beyond the early postoperative period. In addition, maintenance of reduction depends on continued balloon support during the initial healing period, and there is a theoretical risk of partial loss of reduction following balloon deflation and removal. The technique is also highly dependent on appropriate case selection and surgeon experience. Some parameters such as intraoperative ease of the procedure were based on subjective assessment and may be influenced by operator bias. Future studies with larger sample sizes, longer follow-up, and multicentric designs are recommended to further validate the effectiveness of this minimally invasive technique in the management of selected ZMC fractures.

## Data Availability

The original contributions presented in the study are included in the article/Supplementary Material, further inquiries can be directed to the corresponding author.
