# Correction: Evaluation of treatment outcomes of zygomaticomaxillary complex (ZMC) fractures using foley catheter ballooning technique

**DOI:** 10.3389/fdmed.2026.1954964

**Published:** 2026-09-03

**Authors:** Rounak Mukherjee, Tripthi P. Shetty, Padmaraj J. Hegde

**Affiliations:** Department of Oral and Maxillofacial Surgery, AB Shetty Memorial Institute of Dental Sciences (ABSMIDS), Nitte (Deemed to be University), Mangalore, India

**Keywords:** anterior wall of maxillary sinus, foley catheter, n-butyl cyanoacrylate glue, ORIF, ZMC fractures

A correction has been made to **Materials and Methods**, *Study design and ethical approval*, Paragraph 1. This sentence previously included an incorrect Ethics approval number:

“Ethical approval was obtained from the Institutional Ethics Committee of A.B. Shetty Memorial Institute of Dental Sciences, Nitte (Deemed to be University), Mangalore, India (Approval No.: EHICS/ABSMIDS/217/2022).”

The corrected sentence appears below:

“Ethical approval was obtained from the Institutional Ethics Committee of A.B. Shetty Memorial Institute of Dental Sciences, Nitte (Deemed to be University), Mangalore, India (Approval No.: ETHICS/AB SHETTY MEMORIAL INSTITUTE OF DENTAL SCIENCE/431/2024).”

A correction has been made to **Materials and Methods**, *Study design and ethical approval*, Paragraph 1. This sentence previously stated:

“This prospective, randomized comparative clinical study included 42 patients diagnosed with zygomaticomaxillary complex (ZMC) fractures requiring open reduction and internal fixation (ORIF).”

The corrected sentence appears below:

“This prospective, comparative clinical study included 42 patients diagnosed with zygomaticomaxillary complex (ZMC) fractures requiring open reduction and internal fixation (ORIF).”

An incorrect ORCID was linked to author Tripthi P. Shetty. The corrected ORCID link has been corrected to orcid.org/0000-0002-3614-5709.

The original version of this article has been updated.

